# A case of peripheral and eosphagatic hypereosinophilia and food wllergy after liver’s transplantation in adult

**DOI:** 10.1186/2045-7022-1-S1-P87

**Published:** 2011-08-12

**Authors:** Paola Minale, Elena Penza, Susanna Voltolini, Donatella Bignardi, Paola Dignetti

**Affiliations:** 1 St Martino Hospital, Allegy Unit, Genoa, Italy; 2 ASL22, AL, Italy, # Emergency Dept, Villa Scassi Hospital, Allergy Dept, Genoa, Italy

## 

From one year of liver's transplantation and cyclosporin (CSA) maintenance treatment(100 mg a day), a 43 years old women presented with abdominal pain and diarrhea immediately after eating milk and egg. She underwent the transplantation after an acute liver toxicity caused by isoniazid during tuberculosis treatment. Increasing hyperosinophilia (40%) and eosinophilic oesophagitis (EE) presented with food allergy. Total IgE were low (45UI).

Commercial skin prick test and prick by prick as specific IgEs detection (IMMUNOCAP) were positive for milk's proteins, egg, rice and wheat flour. Hypereosinophilia persisted inspite of an elimination diet for the culprit allergens while the clinical symptoms of food allergy improved.

Other causes of hypereosinophilia were excluded.

Interestingly only an inhaled fluticasone propionate treatment (FP,250 mcg bid) for three months was followed by an outstanding reduction of EE and hypereosinophilia (less than 1000/mm3 and <25%) without changing CSA dosage. Food allergy and hypereosinophilia has been increasingly reported in children in the setting of liver transplantation during tacrolimus treatment.On the contrast reports in adults are very rare expecially during CSA treatment. In our patient elimination diet wasn't followed by a reduction of hypereosinofilia as generally occurs in pediatric cases. Our patient didn't present any allergy before the transpantation.No information was available on donor's known allergy.

Different meccanisms are supposed underlying the new onset of food allergy and hypereosinophilia in liver's transplantation:

• An imbalance between Th1/Th2 cells or an increased enteric permeability.

• Immune effects of viral infections associated with the immunosoppressive state.

• Acquired food allergy and hypereosinophilia due to a transfer of hepatic hematopoietic stam cells or active IGE from the donor's liver.

More studied are needed in a controlled setting to identify similar findings among liver transplants.Moreover in the pretransplant investigation should be inlcluded the allergic status both of the donor as the recipient.

